# Simple urine storage protocol for extracellular vesicle proteomics compatible with at-home self-sampling

**DOI:** 10.1038/s41598-021-00289-4

**Published:** 2021-10-21

**Authors:** L. A. Erozenci, T. V. Pham, S. R. Piersma, N. F. J. Dits, G. W. Jenster, M. E. van Royen, R. J. A. Moorselaar, C. R. Jimenez, I. V. Bijnsdorp

**Affiliations:** 1 Department of Urology, Cancer Center Amsterdam, Cancer Center Amsterdam, Amsterdam UMC, de Boelelaan 1117, 1081 HV Amsterdam, The Netherlands; 2 Department of Medical Oncology, Cancer Center Amsterdam, Amsterdam UMC, OncoProteomics Laboratory, de Boelelaan 1117, 1081 HV Amsterdam, The Netherlands; 3 Department of Urology, Erasmus MC, Wytemaweg 80, 3015 GE Rotterdam, The Netherlands; 4 Department of Pathology, Erasmus MC, Wytemaweg 80, 3015 GE Rotterdam, The Netherlands

**Keywords:** Biomarkers, Oncology

## Abstract

Urinary extracellular vesicles (EVs) have gained increased interest as a biomarker source. Clinical implementation on a daily basis requires protocols that inevitably includes short-term storage of the clinical samples, especially when collected at home. However, little is known about the effect of delayed processing on the urinary EVs concentration and proteome. We evaluated two storage protocols. First, urine stored at 4 °C. Secondly a protocol compatible with at-home collection, in which urine was stored with the preservative EDTA at room temperature (RT). EVs were isolated using the ME-kit (VN96-peptide). For both conditions we explored the effect of storage duration (0, 2, 4 and 8 days) on EV concentration and proteome using EVQuant and data-independent acquisition mass spectrometry, respectively. The urinary EV concentration and proteome was highly stable using both protocols, in terms of protein number and quantitative changes. Furthermore, EDTA does not affect the urinary EV concentration or global proteome. In conclusion, urine can be stored either at 4 °C or with EDTA at RT for up to 8 days without any significant decay in EV concentration or a notable effect on the EV-proteome. These findings open up biomarker studies in urine collected via self-sampling at home.

## Introduction

Urine is an advantageous biofluid and a rich source of biomarkers. Its collection is non-invasive, large volumes can easily be obtained several times per day, and it can even be collected at home. Currently, urine is used to diagnose urogenital diseases, such as bladder infections or kidney damage, but can also be used to assist in the diagnosis of prostate cancer^1^. Several disease biomarkers were identified in in urine, including proteins^2–4^. However, urine also contains proteases, such as matrix metallopeptidases, aminopeptidases and cathepsins that degrade (soluble) proteins^5, 6^. Urinary extracellular vesicles (EVs) provide an alternative and valuable resource for protein biomarkers as they offer a protective environment for their cargo due to their bi-layer membrane^7, 8^. Therefore, the urinary EV proteome is expected to be relatively stable after its collection^8^.

EVs are biomarker-rich organelles because their content, which consists of proteins, nucleic acids and lipids among other biomolecules, represents largely their cell-of-origin^9^. They are secreted by almost every tissue type into the biofluids including urine and blood, with over 10^9^ vesicles/ml^10^. Specific markers from distant organs have been reported to be present in urine^11, 12^. Furthermore, fluorescently labeled EVs that were injected into the blood could detected in urine, indicating that EVs can pass the kidneys^13^. EVs play major roles in the biology of various diseases. When cellular proteomes are altered upon disease development, such as cancer, the EV cargo is also changed^14^. Mounting evidence indicates that urinary EV proteins can be used to detect different diseases, including proximal cancers such as prostate and bladder, and also distant diseases such as Alzheimer’s, Parkinson or lung cancer^2, 4, 15–17^.

Depending on several factors such as location of collection (clinic versus home), time/day of collection, and distance to the biobank, urine is not processed immediately after collection, but usually stored for minutes to hours until processing. Urine is preferably kept at 4 °C until bio-banking as it is known that higher temperatures and longer storage may influence the yields of EV-associated biomolecules and EV morphology^18^, as well as decrease the number of urinary EVs^19^. Moreover, bacterial growth can form in urine stored at RT^20^, which might lead to temperature- and time-dependent changes in the urinary EV concentration, as well as the proteome profile^5, 6^. When urine is collected at home, it may even take up to one week until the sample arrives in the laboratory. To reduce these detrimental effects, urine is frequently stored at 4 °C for a maximum of 8 h^21–23^. Previously, we demonstrated that addition of EDTA for storage at RT or storage at 4 °C without preservative demonstrated to inhibit the growth of bacteria, and also it had no effect on (methylated)-DNA levels in urine^24^. However, little is known about the stability of the urinary EV proteome after its collection in a setting of delayed processing and freezing.

In the current study, we examined urinary EV concentration and proteome after short-term storage at 4 °C or at RT with EDTA. To this end, we employed mass spectrometry-based proteomics of urinary EVs. We show that urinary EV concentration and proteome is highly similar in all conditions, showing no clear difference when stored for up to 8 days at RT with EDTA or at 4 °C. These protocols show that urine can easily be self-sampled at home by patients, compatible with EV protein biomarker research.

## Materials and methods

### Urine collection and storage

**Figure 1:**
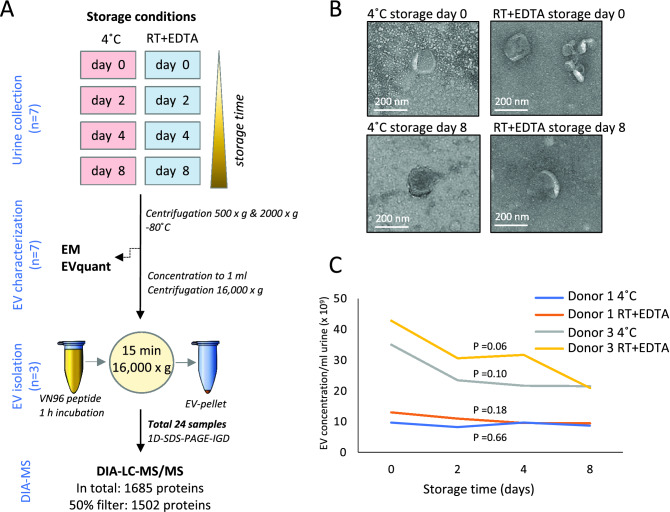
Schematic overview of urine storage conditions. (**A**) Schematic overview of the two storage methods of urine from seven different donors. Urine was stored either at 4 °C or with EDTA at RT for up to 8 days. EV characterization was performed on all seven donors using EM and EVQuant. Urinary EVs were isolated from three donors and proteomes were measured using DIA-LC–MS/MS. (**B**) EM pictures of EVs directly after urine collection, or after 8 days of storage using the two different storage conditions. (**C**) Effect of storage time and protocol on the concentration of EVs as measured using the EVQuant assay.

The study was approved by the Amsterdam University Medical Center (Amsterdam UMC, Location VUMC) local Medical Ethical Committee (METC reference number #2019.443). Urine was collected after a signed informed consent was obtained from each participant. All methods and experiments were performed in accordance with the relevant guidelines and regulations, which is in accordance with the Declaration of Helsinki. Urine was collected from seven different anonymous healthy donors and was aliquoted to eight equal volume fractions from each donor. EDTA was added to a final concentration of 40 mM to 4 of the fractions. Samples were immediately processed (t = 0) or either stored at 4 °C without preservative or at room temperature (RT) with EDTA for 2, 4 or 8 days. After these short-term storage intervals, urine was stored in the biobank at − 80 °C after pre-centrifugation at 500×*g* for 10 min and 2000×*g* for 20 min (See Fig. 1A for a schematic overview of the protocol)^7, 25^.

### EV isolation

Urinary EVs were isolated from 7.5 ml urine as described previously^25^. Nonidet™ P 40 (0.05%, Sigma-Aldrich, Zwijndrecht, The Netherlands) and a protease inhibitor cocktail (PIC, #10276200, Roche) were added to each sample, and the samples were concentrated to 1 ml using a 100 kDa-cutoff filter (Merck Millipore, Billerica, MA, United States). Subsequently urine was centrifuged for 15 min at 16,000×*g* at 4 °C. VN96-peptide (40 µl, Microvesicle Enrichment kit, New England Peptide, #W1073-2, USA) was added, and samples were incubated on a rotation wheel for 1 h at RT. EVs were pelleted by centrifugation for 15 min at 16,000×*g*. The pellet was washed in PBS-containing PIC, and EVs were collected after 16,000×*g* centrifugation at 15 min in LDS-sample buffer containing 10% Dithiothreitol (DTT) (Life Technologies, Carlsbad, CA, cat No:NP0008).

### EV quantitation

EVs in the urine samples were quantified with the EVQuant assay as described previously^26^. In short, EVs in 40 µl unprocessed urine were fluorescently labeled with the generic Octadecyl Rhodamine B Chloride (Rhodamine-R18) membrane dye (Life Technologies) for 10 min. The fluorescently labeled EVs are mixed with a non-denaturating polyacrylamide gel solution and transferred to a glass bottom 96-well plate (P96-10-0-N, Cellvis). After polymerization of the gel, the immobilized EVs were automatically imaged in 25 imaging fields using an Opera Phenix spinning disk confocal HCS (Perkin Elmer) equipped with a 40× water immersion objective (NA 1.1). The Rhodamine-R18 signals of the individual EVs were imaged using the 561 nm laser line and 570–630 nm emission filter and detected using a custom peak detection protocol in the Harmony analysis software (version 4.9, Perkin Elmer). A Rhodamine-R18 dye only control and a 100 nm liposome sample were included to determine background levels and as standard controls. After background subtraction, the EV counts in the calibrated imaging volume were converted to EV concentration.

### Transmission electron microscopy

For transmission electron microscopy, 10 µl unprocessed urine sample were applied to a Formvar/Carbon coated 400 Mesh Cu grid (Van Loenen Instruments) and incubated for approximately 10 min to allow the EVs to adhere to the grid. Excess liquid was carefully absorbed using filter paper. EVs were negatively stained using Uranyless EM stain (Electron Microscopy Science) for 1 min after which the excess Uranyless stain was carefully absorbed using filter paper and the grid was air dried. The EV loaded grids were stored at room temperature in a clean container to avoid dust. The EVs loaded grids were imaged using the Talos L120C TEM (FEI) equipped with a 4 × 4 K CMOS camera at an operating voltage of 120 kV.

### DIA LC–MS/MS proteomics

For proteomics of the 24 individual samples of the current study, each sample was loaded on gradient gels from Invitrogen (NuPAGE 4–12% Bis–Tris gel, 1 mm × 10 wells). The gels were stained with Coomassie brilliant blue G-250 (Pierce, Rockford, IL), reduced by 10 mM DTT/50 mM ammoniumbicarbonate (ABC) at 56 °C for 1 h and alkylated with 50 mM iodoacetamide/50 mM ABC at room temperature (RT) for 45 min. After washing sequentially with ABC and ABC/50% acetonitrile (ACN), the whole gel lanes were sliced in three bands per sample. Gel parts were sliced into cubes of 1 mm^3^, which were incubated overnight with 6.25 ng/mL trypsin (Promega, sequence grade V5111). Peptides were extracted once in 1% formic acid and twice in 5% formic acid/50% ACN. The extracts of three bands were pooled per sample, to obtain one single LC–MS/MS measurement per individual sample. The volume was reduced to 100 µl to remove the acetonitrile in a vacuum centrifuge at 50 °C and samples were desalted using a 10 mg OASIS HLB column (Waters, Milford), after acidification with 0.1% Trifluoroacetic acid (TFA). Samples were eluted in 80% ACN/0.1% TFA and were dried in a vacuum centrifuge. Peptides were redissolved in 20 µl loading solvent (4% ACN in 0.5% TFA) for LC–MS analysis. Peptides were separated by an Ultimate 3000 nanoLC system (Dionex LC-Packings, Amsterdam, The Netherlands), equipped with a 50 cm × 75 µm ID nanoViper fused silica column packed with 1.9 µm 120 Å Pepmap Acclaim C18 particles (Thermo Fisher, Bremen, Germany). After injection, peptides were trapped at 3 μl/min on a 10 mm × 100 μm ID trap column packed with 3 μm 120 Å Pepmap Acclaim C18 at 0% buffer B (buffer A: 0.1% formic acid in ultrapure water; buffer B: 80% CAN + 0.1% formic acid in ultrapure water) and separated at 300 nl/min in a curved 10–52% buffer B gradient in 120 min (140 min inject-to-inject). Eluting peptides were ionized at a potential of + 2 kVa into a Q Exactive mass spectrometer (Thermo Fisher, Bremen, Germany). Data was measured using a data-independent acquisition (DIA) protocol. The DIA-MS method consisted of an MS1 scan from 350 to 1400 m/z at 120,000 resolution (AGC target of 3E6 and 60 ms injection time). For MS2, 24 variable size DIA segments were acquired at 30,000 resolution (AGC target 3E6 and auto for injection time). The DIA-MS method starting at 350 m/z included one window of 35 m/z, 20 windows of 25 m/z, two windows of 60 m/z and one window of 418 m/z, which ended at 1400 m/z. Normalized collision energy was set at 28. The spectra were recorded in centroid mode with a default charge state for MS2 set to 3+ and a first mass of 200 m/z. A spectral library was generated from pooled isolated urinary EVs from eight different anonymous healthy donors, of which four female and four male donors. Thermo DIA raw files were searched in Spectronaut version 13.10 (Biognosys, Schlieren, Switzerland) with default settings. The search result was exported at the fragment ion level for MaxLFQ protein quantification^27^.

### Data analysis and statistics

Analysis was performed in R version 4.0.2 (https://www.R-project.org/). Heatmaps were made using Complex Heatmaps version 2.5.5^28^. Spearman correlation analysis was performed using the corrplot-package version 0.84, with order “hclust” and method “median”^29^. Correlation analysis was performed using Rho-correlation. The group fold change is calculated as exponentiation of the difference of the means of the two groups in log space. Statistical differences between EDTA compared to 4 °C stored samples was performed using Student’s T-test. Venn diagrams were made using Venny 2.1.0^30^. p-values < 0.05 were considered to be significant.

## Results

### Urinary EV concentration, morphology and proteome is highly stable during storage for up to 8 days

We investigated the effect of two short-term urine storage protocols on the EV quantity and the global urinary EV proteome. One protocol included the addition of a preservative EDTA to allow urine to be stored at RT (RT + EDTA), and the other without preservative and stored at 4 °C. Urine was collected from three different donors, one female (donor 1) and two males (donors 2 and 3), on which EV characterization and downstream proteomics was performed. In addition, four independent samples (one male (Donor 4) and three females (Donor 5, 6, 7)) were additionally collected for EV characterization. Urine was processed directly, or after storage for 2, 4, or 8 days (Fig. 1A). Preliminary EM analysis revealed that limited number of EVs can be observed in unprocessed urine samples (i.e. no EVs were isolated). From the few observed EVs in the EM images, no clear change in morphology could be observed between 0 and 8 days, neither stored at 4 °C nor at RT + EDTA (Fig. 1B and Suppl. Figure 1A). To examine the effect of short-term storage at 4 °C or at RT + EDTA on EV quantity, EVQuant was performed on all collected urine samples to measure the concentration of urinary EVs in time. Storage for up to 8 days did not significantly affect the EV concentration in urine, although the EV concentration seems to be slightly decreased in donor 3 (Fig. 1C and Suppl Figure 1B). While the EV concentration varied somewhat between the different donors, storage for up to 8 days did not show a clear change on EV concentration within all tested donors (Fig. 1C and Suppl Figure 1B).

**Figure 2:**
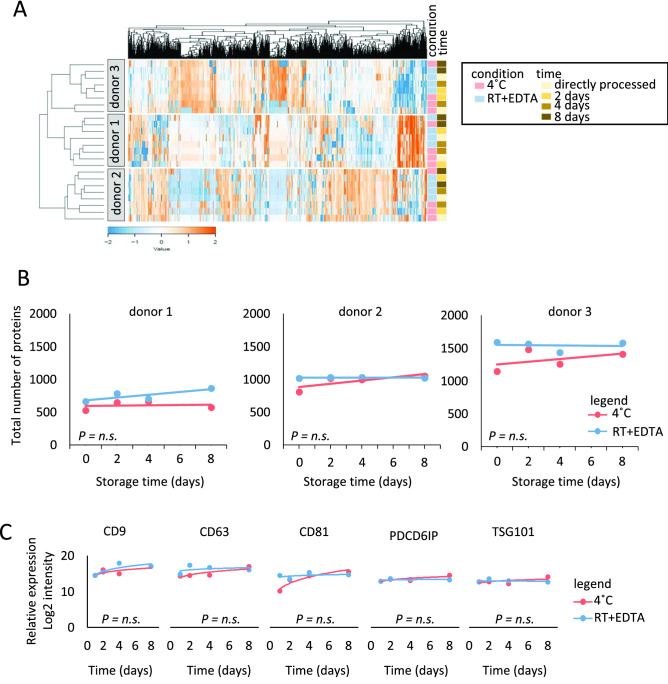
Effect of short-term urine storage on EV proteins. (**A**) Unsupervised heatmap of the urinary EV proteins identified in all samples, showing a clear donor-specific urinary EV profile, and no clustering between urine samples stored at different timepoints, and also not when stored at RT + EDTA or at 4 °C. (**B**) Total identified proteins over time does not show a significant difference in all three donors, under both storage methods. (**C**) Abundancy of several EV markers over time does not show a significant change in donor 2, under both storage conditions (abundancy of several EV markers over time in all donors is show in in Suppl. Figure 1B).

To evaluate whether the urinary EV proteome was affected after the short-term storage, we employed protein profiling using DIA-MS. In total, 1685 proteins were identified using a dedicated spectral library consisting of urinary EV proteome data^31^. To focus on robustly identified proteins, a 50% overall data-presence filter was applied, resulting in 1502 proteins. No consistent pattern was observed in storage time-dependent clustering within each donor (Fig. 2A), indicating that short-term storage time (e.g. within 8 days) is not of clear influence on the global urinary EV proteome using both protocols. Unsupervised hierarchical cluster analysis revealed mainly donor-specific profiles, underscoring the unique protein expression patterns in each donor (Fig. 2A). This personal urinary EV proteomes of different individuals was also observed previously^31^. There was a slightly higher number of proteins identified in some samples in the time course (e.g., day 8 sample donor 1 at the 40C condition and day 2 sample of donor 3 at the RT condition) (Fig. 2B), with no consistent pattern in these minor fluctuations that may be the result of slight differences in the LC–MS or data analysis. Further examination of the possible effect of short-time storage on the urinary EV proteome showed no significant change in the number of total identified proteins across the four timepoints (up to 8 days) for all three donors (Fig. 2B), neither when stored at RT + EDTA nor when stored at 4 °C. No effect of time on the expression of known EV markers (CD9, CD63, CD81, PDCD6IP and TSG101) was observed, indicating that the urinary EV-associated proteins are highly stable (Fig. 2C and Suppl. Figure 2A).

To determine which proteins were significantly changed after 2, 4 and 8 days, statistical analysis was performed for each time separately compared to t = 0. In samples stored at 4 °C, only 72, 94 and 60 proteins were significantly (p < 0.05) changed at 2,4 and 8 days compared to t = 0, of which 72, 30 and 31 were decreased more than twofold. Venn analysis of the overlapping significant proteins revealed only 4 proteins that were changed at all time points (Suppl. Figure 2B). All four proteins were downregulated, of which two proteins were decreased > twofold at all time points, including MCH1 and CA1. In samples stored at RT + EDTA, less proteins were significantly changed compared to 4 °C (Suppl. Figure 2C). After 2, 4 and 8 days of storage, 48, 33 and 51 proteins were significantly (p < 0.05) changed compared to t = 0, of which 14, 5 and 19 were decreased more than twofold. Venn analysis of the overlapping significant proteins revealed no proteins that were changed (both up and down) at all time points (Suppl. Figure 2C).

**Figure 3:**
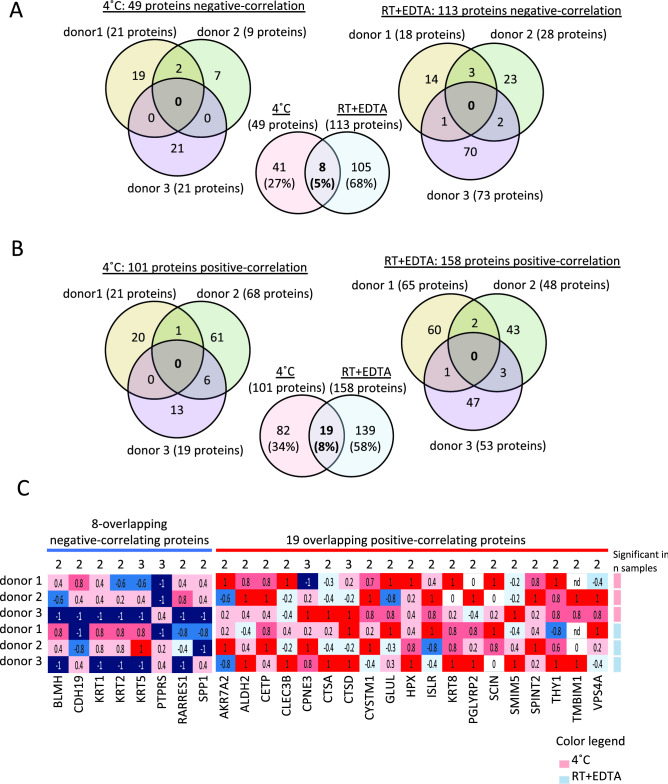
Time dependent effect of short-term urine storage on the global urinary EV proteome. (**A**) Venn diagrams showing proteins that are showing a significant negative correlation (Rho-correlation analysis) over time between the three donors for samples stored at 4 °C or at RT + EDTA. A limited number of donor-unique proteins that are decreased in time, with only eight proteins in overlap to be downregulated in urine samples stored at 4 °C and at RT + EDTA. (**B**) Venn diagrams showing proteins that are showing a significant positive correlation over time (Rho-correlation analysis) between the three donors for samples stored at 4 °C or at RT + EDTA. Of all positive correlating proteins, 19 were in overlap in urine samples stored at 4 °C and at RT + EDTA. (**C**) Heatmap analysis showing the rho values and the number of samples in which the correlation was found to be significant for the 8 negative and 19 positive correlating proteins. No consistency was observed between the samples and the proteins towards the positive or negative correlation.

To assess which urinary EV proteins might be significantly changed in a time dependent manner, Rho-correlation analysis was performed. Between 9 and 21 proteins were significantly reduced in a time-dependent manner when stored at 4 °C, while for urine stored at RT + EDTA, this was between 19 and 74 proteins per donor (Fig. 3A and Suppl. Table 1). Importantly, down-regulated proteins were donor-specific and are not linked to specific protein classes. Only eight of these proteins were in overlap between RT + EDTA and 4 °C storage (Fig. 3A–C, Suppl. Table 1). Between 19 and 68 proteins showed a positive correlation when stored at 4 °C, and between 48 and 65 proteins when stored at RT + EDTA (Fig. 3B). Of these proteins, 19 were in overlap between RT + EDTA and 4 °C storage. To evaluate the relevance of these positive and negative correlating proteins, heatmap analysis was performed from the 8 and 19 overlapping proteins (Fig. 3C). Importantly, most of the proteins that are identified in both 4 °C and RT + EDTA samples are not positive or negatively regulated across all samples. Even though the correlation was significant, the fold in- or decrease of these proteins was low for each of the observed proteins (Suppl. Figures 3 and 4). Altogether, these results indicate that only a limited number of proteins are affected by short-term storage at RT + EDTA or at 4 °C and that these are mostly donor-dependent.

### EDTA does not affect urinary EV concentration, morphology or proteome profile

**Figure 4:**
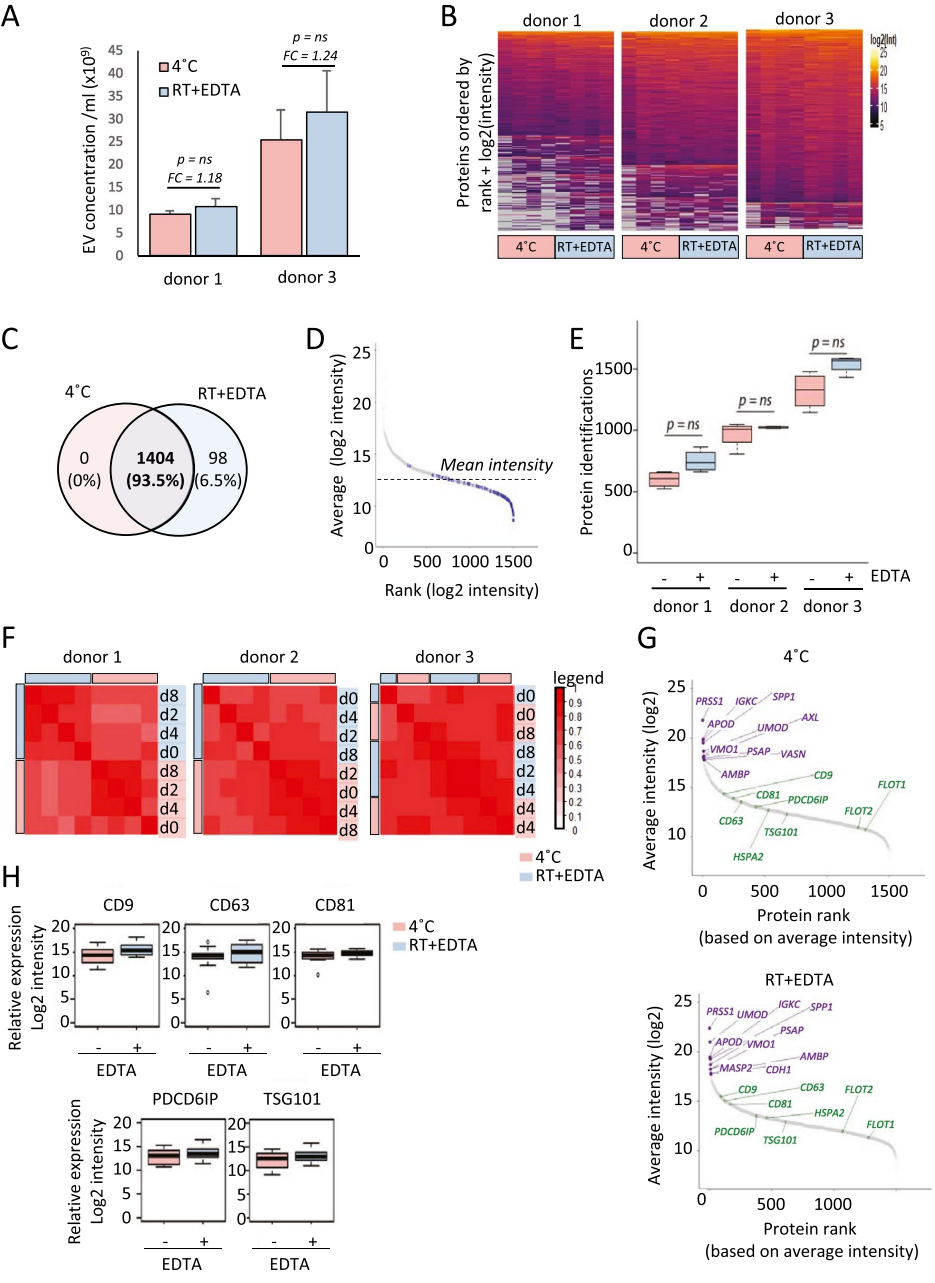
Effect of EDTA on urinary EV proteins. (**A**) Effect of EDTA on the concentration of EVs as measured using the EVQuant assay. (**B**) Data presence plot, showing a high data presence amongst all samples. The proteins were ranked according to data presence and average log2-intensity. The missing values are gray. (**C**) Venn diagram showing the number of proteins identified in urine samples stored at 4 °C vs EDTA. (**D**) Protein rank plot showing that the 98 unique proteins identified upon addition of EDTA (indicated in blue) are low abundant. (**E**) Number of proteins identified in the three donors with and without EDTA addition, showing no significant difference between the three donors. (**F**) Pierson correlation plot showing a high correlation between all urine samples. Blue bars are samples where EDTA is added, pink bars indicate the samples stored at 4 °C. (**G**) Protein rank plot showing that the proteins identified and EV markers are highly consistent. (**H**) Boxplots of known EV markers showing no difference in abundancy between the two storage methods.

To evaluate whether the urinary EVs were affected by EDTA, we also compared urine samples at t = 0 with EDTA to samples where EDTA was not added. Importantly the EV morphology was not clearly affected by adding EDTA, compared to samples where EDTA as not added, as indicated by preliminary EM analysis (Fig. 1B, Suppl. Figure 1A). In addition, no conclusive effect of EDTA addition was observed on the concentration of urinary EVs (Fig. 4A, Suppl. Figure 1C).

To evaluate whether EDTA affected the urinary EV profile, the proteomics data was analyzed. Heatmap analysis shows that urine samples stored with EDTA at RT or at 4 °C were intermixed within the donors, with no clear systematic clustering per method nor time, suggesting that both methods are suitable for urinary EV proteomics and that EDTA does not have a large impact on the overall urinary EV proteome (Fig. 2A). Interestingly, urine samples in which EDTA was added have slightly fewer missing values (average of 144 per paired-sample) compared to urine samples stored without any preservative at 4 °C (Fig. 4B). When EDTA was added, the total protein number was slightly higher (1.14 fold, p = 0.021) compared to samples stored at 4 °C. Of the 98 proteins that were uniquely identified in EDTA-added samples (Fig. 4C) ~ 93% belongs to the 50% of all the proteins that were detected at lower levels (Fig. 4D). Donor 1 had lower number of proteins identified compared to the other donors (Fig. 4E), with about threefold less proteins compared to donor 3, which is consistent with the number of EVs detected in the urine samples (Fig. 4A). In samples where EDTA was added, 92 proteins were significantly changed compared to samples stored without EDTA (t = 0). Of these 92 proteins, 57 were significantly (p < 0.05) increased more than twofold, and 7 were decreased more than twofold (Suppl. Table 2). Amongst 57 the increased proteins, three heat-shock-protein 90 family members were present, including HSP90B1, HSP90AB1, HSP90AA1. When subsequently examining the number of proteins identified between storage at all timepoints without EDTA (at 4 °C, t = 0–2–4–8) and with EDTA (at RT, t = 0–2–4–8), again no significant difference was observed (Fig. 4E).

Because the total number of identified proteins was highly different between the three donors, the effect of EDTA and storage in time per donor was also analyzed after the application of a mild filtering (50% data presence filter) for each donor per condition. This resulted in an average of 678, 996 and 1411 proteins identified for donors 1, 2 and 3, respectively. To examine the comparability of the proteomes between samples with or without EDTA of the three donors, Spearman correlation analysis was performed. All samples highly correlated with Spearman rho values ranging from 0.6 to 0.93 (Fig. 4F). For donors 1 and 2 there seemed to be a hierarchical separation between samples stored with EDTA or at 4 °C, while for donor 3 this was not observed. The top ten abundant urinary EV proteins were highly similar between urine samples stored at 4 °C and with EDTA (Fig. 4G), as well as the levels of known EV markers, including CD9, CD63, CD81, PCDC6IP, TSG101 (Fig. 4H). Taken together, addition of the preservative EDTA to urine does not affect urinary EV concentration, morphology, nor the proteome profile.

## Discussion

Here we demonstrate that urinary EVs keep their integrity and stability when stored either at 4 °C or at RT with the addition of the preservative EDTA for up to 8 days, with no clear effect of storage time nor the temperature on the urinary EV concentration or urinary EV proteome profile. Both RT + EDTA and 4 °C protocol exhibit similar EV concentration and number of identified proteins, hence both methods are suitable for urine storage and downstream urinary EV analysis. Storage at RT (with EDTA addition) allows for flexibility in sample collection, and therefore might be the more convenient protocol.

EDTA is an inexpensive chelating agent that can be used for preservation of urine during storage. Previously, we and others demonstrated the effect of EDTA addition to urine on (methylated) DNA^20, 32^. In these studies, DNA was stable (degradation < 2%) for up to 7 days when EDTA was added, and importantly the EDTA did not influence downstream analysis of DNA using RT-PCR^24^. Although the sample size of three donors is relatively small, the results are highly consistent across the three donors. Whether this consistency is as similar for proteins that are enriched in patients with a disease needs to be investigated in future studies. Importantly, the 76–100% of previously reported core urinary EV proteome of 517 proteins^31^ was identified in our dataset, further demonstrating that the core urinary EV proteome is highly stable when urine has been stored for up to 8 days before freezing in the biobank. In conclusion, we show that urine stabilized with EDTA kept at RT and/or storage at 4 °C both can preserve urinary EV proteins without loss of quality when compared to immediate processing.

Storage of urine at RT with the addition of EDTA is especially preferred when the collection is performed at home, where storage at 4 °C is not possible. Furthermore, there might always be a delay between the collection of urine and its temporary storage into the fridge, even when collected in the hospital. Therefore, we recommend to add EDTA to the urine immediately. This can be also be done by already adding EDTA to the urine collection cup. Importantly, both short-term storage protocols are applicable both to DNA and protein research^24^, therefore, these methods both can be used for multi-omics downstream analyses.

In conclusion, short-term urine storage up to 8 days at 4 °C or with EDTA at RT can be used for EV-proteomics biomarker research. Furthermore, in the current study we measured the urinary proteome in only 7.5–12.5 ml of urine, underlining the clinical potential of DIA-MS-based proteomics. Adding EDTA to urine is recommended to provide a better certainty in the collection and storage quality of urine samples.

## Supplementary Information

Supplementary Information.

Supplementary Table 1.

Supplementary Table 2.
