# Supplementary material for: Simple urine storage protocol for extracellular vesicle proteomics compatible with at-home self-sampling
Source: Sci Rep. 2021 Oct 21;11:20760. doi: 10.1038/s41598-021-00289-4 (PMC8531010; doi:10.1038/s41598-021-00289-4)

SUPPLEMENTAL INFORMATION CAPTIONS

Supplementary Figure 1 – Effect of short-term urine storage on EV number in urine

A. EM pictures of EVs of directly after urine collection, or after 8 days of storage using the two different storage conditions.

B. Effect of urine storage time and protocol on the concentration of EVs as measured using the EVQuant assay.

C. Effect of EDTA on the concentration of EVs as measured using the EVQuant assay.

Supplementary Figure 2 – Effect of short-term urine storage on urinary EV proteome

A. Abundancy of several EV markers over time does not show a significant difference in all three donors, under both storage conditions.

B. Venn diagram showing the number of decreased proteins in urine samples stored at 4˚C for 2, 4 or 8 days compared to t=0.

C. Venn diagram showing the number of decreased proteins in urine samples stored at RT+EDTA stored for 2,4 of 8 days compared to t=0.

Supplementary Figure 3

Correlation graphs of the 8 negatively correlating proteins.

Supplementary Figure 4

Correlation graphs of the 19 positive correlating proteins.

Supplementary Table 1 – Overlapping proteins between urine stored with and without EDTA

Supplementary Table 2 – List of differentially expressed proteins upon EDTA addition

Supplementary Figure 1

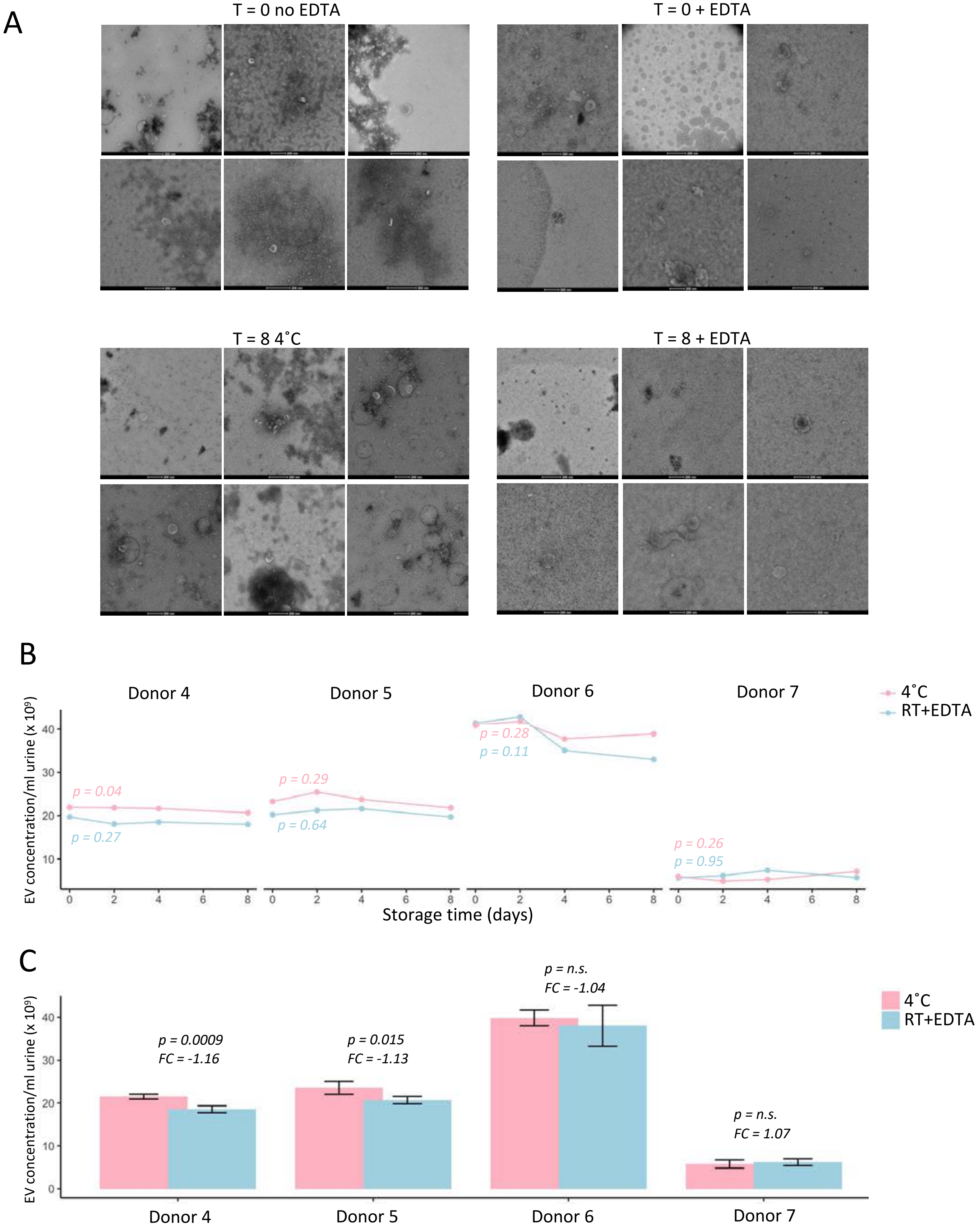

Supplementary Figure 2

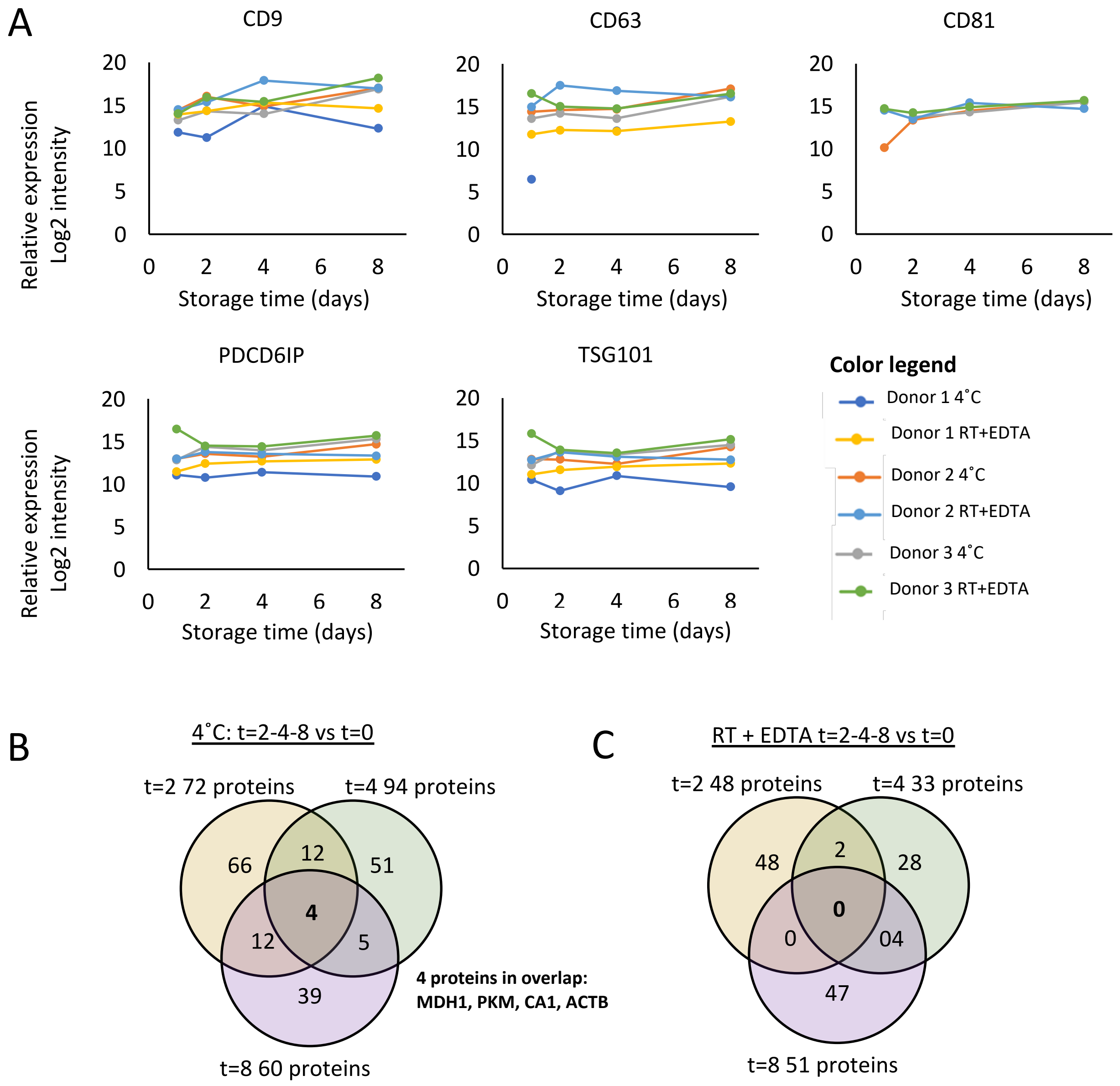

Supplementary Figure 3

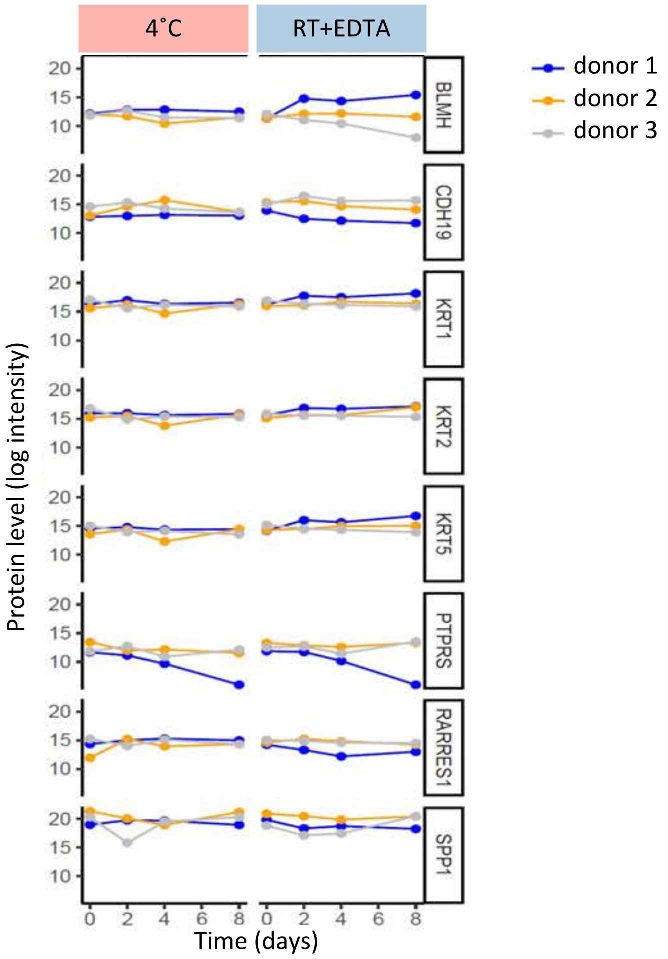

Supplementary Figure 4

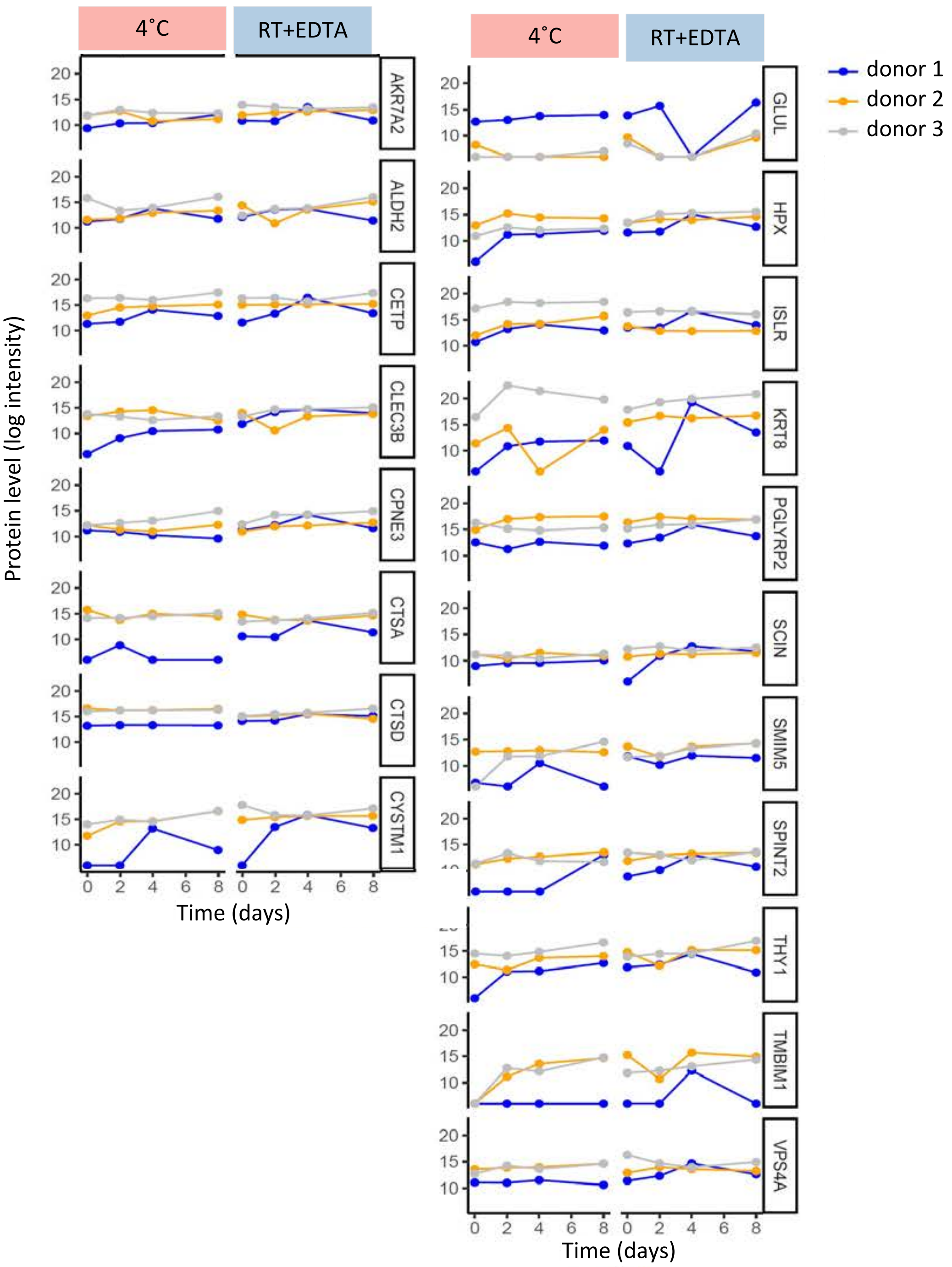

Supplement: Supplementary file 1 — Supplementary Information. [file 41598_2021_289_MOESM1_ESM.pdf]
